# Small Molecules in the Treatment of Acute Severe Ulcerative Colitis: A Review of Current Evidence

**DOI:** 10.3390/ph18030308

**Published:** 2025-02-23

**Authors:** Raffaele Pellegrino, Giuseppe Imperio, Ilaria De Costanzo, Michele Izzo, Fabio Landa, Assunta Tambaro, Antonietta Gerarda Gravina, Alessandro Federico

**Affiliations:** Hepatogastroenterology Division, Department of Precision Medicine, University of Campania Luigi Vanvitelli, Via L. de Crecchio, 80138 Naples, Italy

**Keywords:** acute severe ulcerative colitis, ulcerative colitis, small molecules, tofacitinib, upadacitinib, filgotinib, ozanimod, etrasimod, biologics, sequencing

## Abstract

Ulcerative colitis (UC) is an inflammatory bowel disease in which one-quarter of patients are at risk of developing a severe form of the disease known as acute severe UC (ASUC). This condition exposes patients to serious complications, including toxic megacolon, surgical intervention, and even death. The current therapeutic strategy relies on time-dependent, multi-step algorithms that integrate systemic corticosteroids, calcineurin inhibitors, and biologic agents (specifically infliximab) as medical therapy aimed at avoiding colectomy. Despite this approach, a significant proportion of patients fail to respond to either corticosteroids or infliximab and may require alternative therapeutic options if there is no urgent surgical necessity. These alternatives include other biologics or emerging small molecules, although the evidence supporting these treatments remains extremely low, even considering their well-documented and promising efficacy and safety in moderate-to-severe UC. Conversely, it is necessary to investigate whether infliximab can be effectively replaced or surpassed by other approved biological agents and small molecules as first-line therapy after steroid resistance. This review aims to summarise the available evidence on small molecules, specifically Janus kinase inhibitors and sphingosine-1-phosphate receptor modulators.

## 1. Introduction

Ulcerative colitis (UC) is a chronic, immune-mediated condition with a self-sustaining and relapsing–remitting course, leading to chronic inflammatory damage of the colon and rectum [1]. It is associated with an increased risk of long-term complications, including an elevated risk of colorectal cancer [2].

UC, as an inflammatory bowel disease (IBD), has a rising incidence, estimated annually at 8.8–23.1 per 100,000 person-years in North America and 0.6–24.3 per 100,000 person-years in Europe [3]. The incidence peaks equally between genders during the second to fourth decades of life [3]. With the increasing incidence, global hospitalisation rates are also rising, including in industrialising countries, making this condition a clear epidemiological burden for global healthcare systems [4]. This trend has positioned UC as a global disease, with its prevalence exceeding 0.3% worldwide [5].

The course of the disease is far from predictable, although well-controlled endoscopic activity, achieving even mucosal healing [6], and well-managed faecal calprotectin levels [7] generally suggest a more reassuring disease history. Despite the increasing availability of advanced therapies for UC [8], one-quarter of patients experience at least one episode of severe disease activity (i.e., acute severe UC, ASUC) during their lifetime, and 20% of these patients undergo colectomy during their first hospitalization [9].

However, no randomized placebo-controlled trials are available for ASUC covering all biologics and small molecules approved for use in moderate-to-severe UC, limiting the ability to produce high-level evidence-based clinical practice recommendations for this subset of patients [10]. Moreover, the management of ASUC relies on strictly time-dependent algorithms that weigh the risk of colectomy and the development of high-mortality complications (e.g., toxic megacolon) at each step [11,12]. Therefore, the ability to use rescue therapies following steroid refractoriness (still the cornerstone of ASUC treatment [11,12]) with high speed and efficacy is a significant priority.

Since the late 1990s, with the approval of infliximab (IFX) and subsequent parenteral biologics such as vedolizumab and ustekinumab for UC, new oral molecules (collectively referred to as small molecules) have been developed [13]. These are divided into two main groups: Janus kinase inhibitors (JAK inhibitors) and sphingosine-1-phosphate receptor modulators (S1P modulators) [13]. Moreover, small molecules, particularly JAK inhibitors, have been shown in recent network meta-analyses to perform better than other advanced medical therapies for UC in terms of clinical response, clinical remission, and endoscopic improvement, both in biologic-naïve and biologic-exposed patients [14]. This is especially true regarding speed [14,15], making these molecules highly interesting candidates in the management of ASUC.

Consequently, there is still much to be done in managing ASUC to break through the therapeutic ceiling [16] for UC in this group of high-severity patients. Despite the multiple therapies already approved for moderate-to-severe UC, there is a lack of randomised trials evaluating their efficacy in ASUC, representing the most significant unmet need in ASUC. Steroids still hold an extremely relevant role in ASUC despite their well-known and significant adverse events, particularly in a high-risk surgical setting where perioperative use of steroids increases the risk of postoperative infectious complications [17].

Therefore, there is an imperative need to monitor the emerging evidence for fast-onset drugs, such as small molecules, which hold significant potential in ASUC. This review aims to examine the evidence currently available regarding the use of small molecules in ASUC.

### Search Strategy for This Narrative Review

The identification of the pool of articles to be included in this narrative review was conducted across three main databases using the following detailed search operators:

- MEDLINE: (acute severe ulcerative colitis OR ASUC) AND (tofacitinib OR filgotinib OR upadacitinib OR ozanimod OR etrasimod OR small molecules);
- EMBASE: (‘acute severe ulcerative colitis’ OR ‘ASUC’) AND (‘tofacitinib’ OR ‘filgotinib’ OR ‘upadacitinib’ OR ‘ozanimod’ OR ‘etrasimod’ OR ‘small molecules’);
- Web of Science: ((ALL = (acute severe ulcerative colitis) OR ALL = (ASUC)) AND (ALL = (tofacitinib) OR ALL = (filgotinib) OR ALL = (upadacitinib) OR ALL = (ozanimod) OR ALL = (etrasimod) OR ALL = (small molecules)).

For none of the databases (i.e., MEDLINE, EMBASE, and Web of Science) were additional filters applied for specific study types, languages, or publication years. Moreover, no specific inclusion (or exclusion) criteria were defined for the identified works, as this pertains to a systematic review and falls outside the scope of the present work. The review was conducted using the updates available in the aforementioned databases up to December 2024.

## 2. ASUC: Current Management According to Major Guidelines and Beyond

### 2.1. Current Major Guidelines

ASUC is a form of UC characterised by severe and rapidly progressive disease activity, defined by the Oxford criteria as the combination of ≥ 6 bloody bowel movements per day alongside at least one sign of systemic toxicity, including a body temperature > 37.8 °C, a heart rate > 90 bpm, haemoglobin < 10.5 g/dL, or a C-reactive protein > 30 mg/L [11]. ASUC is a life-threatening condition associated with a mortality risk of 1%, which is higher in older patients [18].

Among ASUC-related complications is toxic megacolon, which places the patient at risk of colectomy [11]. Adherence to the Oxford criteria is associated with an increased risk (OR 4.42) of requiring colectomy within one year, as highlighted in a recent meta-analysis [19].

The current management of ASUC involves diagnostic and therapeutic measures that proceed in parallel [11]. Once the Oxford criteria are met at baseline, it is crucial to initiate infectious disease investigations on stool samples (e.g., to exclude *Clostridioides difficile* superinfection [20]) as well as endoscopic evaluation via flexible sigmoidoscopy (both to assess mucosal disease activity and to obtain biopsies to rule out Cytomegalovirus superinfection [21]). These diagnostic approaches are complemented by therapeutic interventions, including systemic parenteral corticosteroid treatment and prophylactic anticoagulation with heparin [22].

The patient is subsequently monitored for three days to evaluate clinical response (defined as fewer than four bowel movements per day for at least two days without bleeding). If this response is absent, mainly if the patient exhibits more than eight bowel movements per day (or fewer, but with a C-reactive protein >45 mg/L), rescue therapy should be initiated with IFX at 5 mg/kg or ciclosporin at 2 mg/kg [11]. IFX may be repeated at 5 mg/kg on days 6–8 if necessary [11]. Throughout this pathway, the surgeon regularly assesses the potential need for colectomy. Additionally, it is crucial to exclude toxic megacolon when suspected, using radiological tools such as abdominal X-rays or computed tomography.

In addition to the aforementioned British guidelines, the European Crohn’s and Colitis Organisation (ECCO), in its most recent guidelines on ASUC [12], also endorses the use of systemic parenteral steroids as the first-line therapeutic step and IFX or ciclosporin as rescue therapy options. However, it does not provide a strong recommendation regarding the potential use of an accelerated IFX regimen due to a lack of robust evidence [12]. Moreover, the American guidelines also effectively support this approach [23].

The use of an accelerated IFX regimen remains highly debated. A recent meta-analysis involving 2158 ASUC patients reported an overall colectomy-free survival of 79.7% at 3 months and 69.8% at 12 months for those treated with IFX as rescue therapy [24]. Among these, colectomy-free survival at 3 months did not differ significantly between patients who underwent dose intensification using high-dose or accelerated strategies and those who did not [24]. Cyclosporine, a calcineurin inhibitor, on the other hand, has represented a rescue therapy in this specific disease setting since the 1990s [25]. Over time, various comparative studies have supported the growing use of IFX. For example, Laharie et al. [26], in a randomised open-label head-to-head trial between cyclosporine and IFX, suggested a true equivalence between the two treatments. The CONSTRUCT study had previously reported similar data [27].

Moreover, a subsequent randomised controlled trial demonstrated that long-term colectomy-free survival was independent of the initial treatment of steroid-refractory ASUC (whether IFX or cyclosporine) [28]. Tacrolimus, another calcineurin inhibitor, has also been used in the ASUC setting, similarly to cyclosporine [29].

Moreover, a meta-analysis that pooled data from approximately three hundred patients with ASUC, albeit from retrospective evidence, demonstrated an equivalence between IFX and cyclosporine in terms of colectomy rate at 3 and 12 months, as well as in terms of adverse events and postoperative complications [30].

In other words, the standard choice between IFX and calcineurin inhibitors depends on the centre’s experience and local availability.

### 2.2. What Other Biologics-Based Medical Rescue Therapy Options Are Available for Steroid-Refractory ASUC?

Despite the dominance of evidence regarding IFX and cyclosporine as advanced rescue therapies in ASUC, several studies have been conducted on other biologics that have been approved for treating UC.

Vedolizumab, an α_4_β_7_ integrin inhibitor, has not been evaluated in randomised clinical trials specifically for ASUC indications. However, a case report highlighted a positive outcome in a paediatric patient with ASUC who had prior exposure to anti-TNF agents [31]. Additionally, our research group has reported favourable results in a patient with ASUC triggered by chemotherapy in a complex oncological setting where anti-TNF therapy was contraindicated [32]. However, vedolizumab is not a drug associated with rapid induction of clinical remission [33]. Therefore, beyond case reports, the available studies—largely retrospective—have been conducted using a combination of vedolizumab with other agents, predominantly cyclosporine or tacrolimus. A few prospective studies were conducted by Christensen et al. [34] and Tarabar et al. [35] in fewer than thirty patients, where vedolizumab was combined with cyclosporine. Both studies reported a colectomy avoidance rate of 82%. Among the more extensive retrospective studies, Ollech et al. [36] reported on approximately seventy patients, most of whom were bio-experienced with anti-TNF. The colectomy avoidance rate was 58%. However, once again, vedolizumab was combined with another agent that had a faster onset of action (cyclosporine or tacrolimus). Despite the limited evidence, given the impressive safety profile of vedolizumab compared to other approved therapies for UC [37], the prospect of integrating it in combination with another agent with a faster onset of action in ASUC remains intriguing, with vedolizumab serving as the maintenance therapy [38].

The p40 subunit inhibitor of IL-12 and IL-23, ustekinumab, although exhibiting a faster onset of action compared to vedolizumab [15], reflects similar observations based on current evidence. Three studies, primarily case reports or small series (in anti-TNF experienced patients), have demonstrated excellent outcomes with ustekinumab; however, these were combined with cyclosporine and involved fewer than twenty patients [39,40,41].

**Figure 1:**
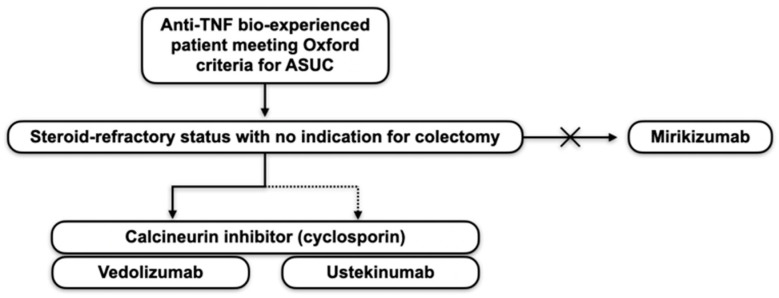
Possible *rescue therapies* with biologics (excluding small molecules) to consider in patients with steroid-refractory acute severe ulcerative colitis (ASUC) without indication for surgery and prior exposure to anti-TNF in the disease history. Given the minimal available evidence, in this setting, a combination approach of ciclosporin and vedolizumab, utilising the faster induction of remission by the former and the potential of the latter as maintenance therapy, could be considered. A similar approach using ustekinumab should be reserved for highly selected patients, likely those who have previously failed even vedolizumab. Currently, there is no evidence to suggest a role for mirikizumab in this subset of patients.

Figure 1 summarises the possible space for current therapies in patients with ASUC who are already experienced with anti-tumour necrosis factor (TNF) agents and steroid-refractory, excluding small molecules.

### 2.3. What Room Is There for Sequencing Rescue Therapy in ASUC?

The failure of a first-line medical *rescue therapy* following steroid-refractoriness—typically, in most cases, the failure of IFX or calcineurin inhibitors—raises the question of whether there is a scope for a second-line rescue therapeutic agent, provided there are no immediate surgical indications.

It should clearly be taken into consideration, as an underlying premise of this reasoning, that these are infrequent cases where there is a rare additional window to sequence multiple rescue therapies one after the other. In such instances, the challenges in gathering robust evidence for ASUC are further reinforced and exacerbated.

In a systematic review, Feuerstein et al. [42] compared four studies employing a third-line therapeutic approach in ASUC, focusing primarily on the use of IFX after cyclosporine failure and vice versa. The analysis revealed no significant differences between these approaches in terms of clinical response, clinical remission, or colectomy rates at twelve months.

More recently, the REASUC retrospective cohort study [43] assessed the safety profiles and colectomy-free survival associated with third-line therapies in patients with steroid-refractory ASUC who had already failed IFX or cyclosporine as second-line rescue therapy (with steroids considered the first-line treatment). Among the 78 patients in the analysis, 80% received either IFX or cyclosporine as the third-line treatment, administered opposite to the agent previously used in the second-line setting. Notably, tofacitinib and ustekinumab were employed as third-line agents in 17% and 3.8% of cases, respectively. The colectomy rate during a median follow-up of 21 weeks was reported at 37%, with an adverse event rate of 33%.

Hassan et al. [44], in a conference abstract, described a small cohort of ASUC patients treated with upadacitinib as a second- or third-line agent following corticosteroid failure. In this series, two patients received upadacitinib as third-line therapy (at a dose of 60 mg per day) after IFX failure, both achieving clinical response in under three days.

Accordingly, third-line therapy should be considered solely for highly selected patients following comprehensive multidisciplinary discussions that carefully weigh the risks and benefits of colectomy, which remains the standard of care in cases of failure to second-line rescue therapy in ASUC [12]. Nevertheless, the progressive integration of new mechanisms of action into ASUC management, guided by emerging evidence, may shed light on their potential positioning beyond second-line therapy in this highly challenging subset of patients. For now, however, the evidence remains critically limited, precluding the adoption of any multi-line sequencing strategy in ASUC within this clinical context [45].

### 2.4. What Are the Limitations of the Current Standard of Care That Highlight the Need for New Mechanisms of Action in ASUC?

IFX, the only biological agent supported by evidence from randomised trials for ASUC [10] and, as previously mentioned, currently the first-line biologic used as rescue therapy in international guidelines, is also the most widely used biologic for moderate-to-severe UC. This is attributable to its historical significance, being the first biologic available for UC and pharmacoeconomic considerations. Indeed, studies examining treatment pathways in IBD have shown that well over half of patients with biologic-indicated UC initiate therapy with an anti-TNF agent, primarily IFX [46]. Nonetheless, it should not be overlooked that approximately 30%—a significant proportion—of patients with ASUC do not respond to treatment with steroids and rescue therapy with IFX [47]. Without urgent indications for colectomy, there may be an opportunity for a second-line rescue therapeutic agent, which must possess characteristics of rapid onset of action.

This creates the issue and high likelihood of encountering, among the 25% of patients with UC who develop ASUC, prior use of IFX and, possibly, a history of primary failure or secondary loss of response (for example, due to the development of anti-drug antibodies). This places the clinician in the dilemma of having to reuse a drug for which the patient has already demonstrated clear inefficacy in the past within an emergency setting where there is little time for therapeutic trials and where even a 1% risk of mortality, alongside a significantly higher risk of other complications, looms, as previously described. In this scenario, which will likely become increasingly representative of both the present and the future, it is imperative to assess the performance of other mechanisms of action that could complement or replace the role that IFX has had and continues to have in ASUC.

However, efficacy rather than effectiveness data are required for a setting derived from randomised controlled trials. Nonetheless, initiating a randomised controlled trial for ASUC presents considerable challenges, including the urgency of treatment, which limits the time available for pre-enrolment screening to assess patients’ eligibility for the trial, as well as the short timeframe for randomisation, increasing the risk of under-recruitment of patients [10]. Moreover, assignment to a placebo group in a situation such as ASUC raises ethical concerns, compounded by significant heterogeneity and difficulties in selecting primary endpoints—due to the lack of clear consensus—between colectomy-free survival and clinical remission [10].

Nonetheless, among the various therapeutic alternatives to anti-TNF agents, drawing from the arsenal of treatments already approved for UC, small molecules offer, unlike other mechanisms previously described, the significant advantage of providing a rapid onset of action, which is undoubtedly essential in ASUC. Based on data from registration trials, as already stated, Panaccione et al. [14] demonstrated in a network meta-analysis that, both in naïve and bio-experienced patients, small molecules (specifically upadacitinib, tofacitinib, and filgotinib) are the most effective in terms of speed of action and achieving clinical response, clinical remission, and endoscopic improvement. In addition to this, preliminary data also suggest that certain small molecules, specifically tofacitinib, are safe in the perioperative setting in terms of potential postoperative complications [48], a relevant finding considering the high surgical risk of ASUC patients.

However, as described below, only data from small initial studies, case reports, or case series are currently available for this type of treatment.

## 3. Small Molecules in ASUC: What Evidence Is Currently Available?

### 3.1. Small Molecules: Classification Principles, Mechanism of Action, and Current Therapeutic Positioning in Ulcerative Colitis

Small molecules are natural or artificial compounds with a molecular weight of less than 1000 Da [49,50]. In contrast to more extensive and heavier biological drugs, which, due to their size, are primarily distributed in the vascular and extracellular compartments, the limited dimensions of small molecules allow them to easily cross the cell membrane, giving them a better and faster diffusion capacity [51]. This results in a rapid onset of action and the ability to modulate intracellular signalling pathways. In contrast, their short half-life makes once- or twice-daily administration necessary [51,52]. In this section, we will briefly describe the molecular mechanisms of action of the two classes of small molecules approved for use in UC: the JAK inhibitors (i.e., tofacitinib, filgotinib, and upadacitinib) and the S1P receptor modulators (i.e., ozanimod, and etrasimod) [53]. Small molecules acting on other molecular mechanisms are under investigation, e.g., phosphodiesterase-4 inhibitors and oral anti-integrins [54].

JAK is a family of tyrosine kinases consisting of four isoforms in mammals: JAK1, JAK2, JAK3, and tyrosine kinase 2 (TYK2) [55]. JAK proteins are associated with the intracellular domain of type I and type II cytokine receptors, thus mediating intracellular signalling of more than 50 cytokines [including interleukins, interferons (IFNs), colony-stimulating factors and growth factors] [56,57]. Their role in response to such a large number of cytokines makes these proteins crucial in regulating various biological functions, such as cell proliferation and differentiation, apoptosis, and haematopoiesis, and they have important regulatory functions on the immune system [58]. Receptor activation by cytokine binding leads to autophosphorylation and/or transphosphorylation of JAKs, as well as phosphorylation of the receptor. Receptor phosphorylation, in turn, promotes binding and JAK-mediated phosphorylation, the signal transducer and activator of transcription (STAT) family members, designated to signal transduction to the nucleus through activation of the gene transcription process. Seven STAT isoforms (STAT1, STAT2, STAT3, STAT4, STAT5A, STAT5B and STAT6) are known, which interact with the four JAKs (JAK/STAT pathway) depending on the specific cytokine [56,59].

JAK inhibitors (JAKi) prevent JAK activation, thus blocking intracellular signalling of multiple cytokines simultaneously. This makes these drugs significantly different from biologics, which instead can inhibit one or a few cytokines at a time since they only act on the extracellular compartment. Based on their ability to block one or more JAKs, JAKi can be defined as non-selective (or pan-selective), i.e., tofacitinib, and selective, i.e., upadacitinib and filgotinib (both JAK1-selective) [60,61]. Although the new JAK inhibitors are designed to be selective, existing studies have questioned the significance of selectivity in efficacy and mitigating adverse events [62].

S1P is a pleiotropic lipid mediator in the sphingolipid class [63]. These lipid mediators play a key role in lymphocyte egress from lymphoid tissues into the lymph and bloodstream and in angiogenesis and vascular integrity [64,65,66]. Their role in lymphocyte trafficking is determined by the S1P gradient formed between blood and lymph, which are more abundant in S1P and tissues, where S1P concentrations are considerably lower [67]. The balance between S1P production and degradation ensures the maintenance of its concentration gradient. Sphingosine kinase (SphK) 1 and 2 are responsible for the production of S1P through phosphorylation of sphingosine, which is, in turn, produced by ceramidase from ceramide [68]. Degradation is driven by the action of S1P lyase (which irreversibly cleaves S1P into phosphoethanolamine and hexadecenal) or S1P-specific or broad-specific lipid phosphatases [69]. Furthermore, erythrocytes (which contain the specific transporter MFSD2B and lack degradation enzymes) and endothelial cells (which express the specific transporter SPNS2) contribute to high S1P circulating levels acting as reservoirs [70,71,72]. Since they are liposoluble compounds in the blood, approximately 65% of S1P is bound to apolipoprotein M (ApoM)-containing high-density lipoprotein (HLD), and the remaining 35% is bound to albumin [73].

S1P exerts its functions as an autocrine or paracrine mediator by binding to five G-protein-coupled receptors on the cell surface, namely S1PR1, S1PR2, S1PR3, S1PR4 and S1PR5 [74]. S1PR1-3 are widely distributed and have the highest expression levels in the cardiovascular and immune systems, while the expression of S1PR4 and S1PR5 is restricted to the lymphatic and nervous systems, respectively [74]. Furthermore, S1P has been hypothesized to act as an intracellular mediator. However, the intracellular signalling functions of S1P have not been completely elucidated, and many functions previously attributed to its role as a second messenger do not entirely exclude the involvement of S1P receptors [75].

S1PR modulators function as agonists or functional antagonists of S1P [76]. Excluding fingolimod, the first of this class of drugs to be produced, which binds S1PR1, S1PR3, S1PR4 and S1PR5 with high affinity, the more recently produced S1PR modulators manifest greater selectivity; ozanimod is S1PR1- and S1PR5-selective [77], while etrasimod is S1PR1-, S1PR4- and S1PR5-selective [78]. Given their central role in lymphocyte trafficking, blocking S1PR signalling, in particular S1PR1-mediated response, results in the retention of lymphocytes in lymphoid organs, preventing their access to circulation and sites of inflammation [79]. In addition, the anti-inflammatory properties of S1PR agonists might be due to adjunctive potential effects on dendritic cell migration and vascular barrier function [80].

**Figure 2:**
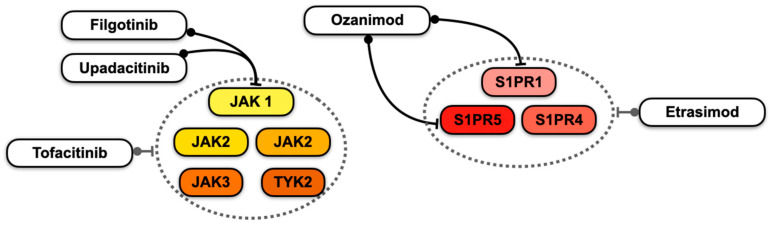
Selectivity profiles of ulcerative colitis approved Janus kinase (JAK) inhibitors, including tyrosine kinase 2 (TYK2), as well as sphingosine-1-phosphate receptor (S1PR) modulators. The dashed circles indicate that the selected molecules (i.e., tofacitinib and etrasimod) inhibit the entire set of targets within the circle.

Figure 2 summarises the selectivity profiles of JAKi and S1P receptor modulators.

Even though all the above-mentioned small molecules have been demonstrated to be effective in UC treatment, the current guidelines of the major international societies only consider using tofacitinib in moderate-to-severe UC but not ASUC. In contrast, the other JAK inhibitors and S1P modulators are not indicated as possible therapies [8,81].

### 3.2. Tofacitinib

Tofacitinib belongs to the small molecule category and has been approved for treating UC. Recently, considerable interest has arisen in the use of tofacitinib as rescue therapy in the management of ASUC. Tofacitinib offers several advantages in the treatment of ASUC. These include its oral administration, high therapeutic efficacy, and rapid clearance from the body, with a half-life of approximately 3.2 h. This rapid clearance may provide a theoretical advantage by minimising the risk of complications during and after surgeries such as colectomy, even in emergencies, ensuring quick drug elimination [82]. Additionally, tofacitinib appears to be less affected by conditions such as hypoalbuminemia [82]. As a small molecule, its efficacy is not significantly reduced by protein loss in the colon, a common issue with biologic drugs [83,84].

Another relevant aspect is its efficacy in steroid-resistant UC. Resistance to these drugs is associated with elevated levels of pro-inflammatory cytokines, such as interleukin IL-6 and IL-8, alongside genetic factors. In vitro studies have shown that IL-2 hinders activation of the glucocorticoid receptor by interfering with its movement into the cell nucleus through a mechanism mediated by phosphorylation of STAT5, under the control of JAK1 and JAK3. By inhibiting the STAT-mediated signalling pathway, tofacitinib may disrupt these processes, restoring corticosteroid responsiveness and offering a potential solution in refractory cases [85,86,87]. A systematic review examined studies evaluating tofacitinib use in ASUC patients, focusing on colectomy-free survival rate. The review included 148 patients treated with tofacitinib after failure of salvage corticosteroid therapy and previous biologic treatments. The data revealed that survival without colectomy was 85% at 30 days, 86% at 90 days and decreased to 69% at 180 days [88]. Standard first-line therapy for ASUC with i.v. corticosteroids results in a 29% colectomy rate. Although tofacitinib is generally used as a last resort in patients with few therapeutic alternatives other than colectomy, results show that it can achieve results comparable to those of traditional therapies [89]. A retrospective case–control study analysed tofacitinib’s effectiveness in inducing remission and reducing colectomy risk at 90 days in ASUC patients previously treated with biologics and corticosteroids [90]. All patients studied received i.v. corticosteroids. In the control group, two patients (1.8%) received cyclosporine as rescue therapy, while 43 patients (38.1%) were treated with IFX. Among them, 13 (11.5%) followed an accelerated protocol involving two doses of IFX administered before the standard dose on day 14. In the group treated with tofacitinib, no patients received IFX or cyclosporine as therapeutic support. Among patients on tofacitinib therapy, 16 (40%) took the drug at a dosage of 10 mg twice daily (BID), while the remaining 24 (60%) received 10 mg three times daily (TID) [90]. The results showed that six patients (15.0%) in the tofacitinib group and 23 (20.4%) in the control group underwent colectomy within 90 days. This finding indicates that the risk of colectomy at 90 days was significantly lower in patients treated with tofacitinib than in controls. Multivariate analysis examining the effect of tofacitinib dosing, considering the “no administration”, “10 mg BID”, and “10 mg TID” categories, revealed that treatment with a dose of 10 mg TID exerted significant protection against colectomy. However, the same protective effect was not observed with the 10 mg BID dosage within 90 days [90]. Similarly, Gilmore et al. [91] reported on five steroid-refractory ASUC patients who were unresponsive to rescue therapy with IFX treated with high-dose tofacitinib (10 mg TID for 14 days). Four achieved clinical response without colectomy, while two achieved clinical and endoscopic remission at 90 days. Only one patient underwent colectomy after clinical worsening at dose reduction. Although small in scale, these results are encouraging and suggest the safety and effectiveness of extended high-dose tofacitinib in refractory cases.

A meta-analysis of 2004 patients across 21 studies compared various treatments for steroid-refractory ASUC [92]. Treatments evaluated included tofacitinib (20 mg daily IFX (induction dose of 5 mg/kg administered at 0, 2 and 6 weeks), IFX 10 (induction dose of 10 mg/kg administered at the exact times), IFX administered in an accelerated regimen (three doses of 5 mg/kg with timings adapted to clinical needs), tacrolimus, cyclosporine, ustekinumab, and adalimumab. These treatments were compared with placebo. Tofacitinib was the most effective in reducing short-term colectomy rates, followed by IFX 10 and tacrolimus. In contrast, colectomy rates observed with ustekinumab and adalimumab were not significantly different from placebo-treated groups [92]. In addition to reducing colectomy rates, tofacitinib showed high clinical and endoscopic efficacy, with 69% achieving clinical remission and 55% endoscopic remission at 90 days [88].

In a case report dating back to 2021, at Lisbon Hospital, two patients received tofacitinib as rescue therapy in the context of ASUC [93]. Both patients had a total Mayo score of 11 and a Mayo endoscopic score of 3 on hospital admission. After 5–7 days of methylprednisolone IV without achieving clinical improvement, tofacitinib was administered at a dosage of 10 mg BID. After 5–7 days of therapy, clinical improvement was observed with reduced daily bowel frequency and C-reactive protein below 10 mg/L. After 8 weeks, the total Mayo score was 3 and 2, respectively, and after 16 weeks, both patients had achieved endoscopic remission with a total Mayo score of 1. After 14 and 11 months of follow-up, respectively, the patients remained in steroid-free remission, avoiding colectomy [93].

The effectiveness of tofacitinib in inducing clinical response was also confirmed in a retrospective analysis in which eight patients with ASUC who had not responded to i.v. hydrocortisone administration for 5–7 days were identified [94]. These patients were subsequently treated with tofacitinib at a dose of 10 mg BID. All eight patients had previously used 5-ASA, four (50%) had experience with azathioprine, and only one had previously received IFX and vedolizumab. After initiation of tofacitinib therapy, seven out of eight patients showed a clinical response, achieved by all within five days of starting treatment. Additional endpoints analysed were steroid-free remission (SFR) rate, primary non-response rate (PNR) and secondary loss of response (LOR). A multicentre retrospective study analysed the use of tofacitinib in different conditions, involving 391 patients with UC with a median observation period of 26 weeks [95]. The study included patients with chronic activity (70.1%), ASUC (27.1%), pouchitis (2.6%), and other unclassified cases (0.2%). All participants received an induction dose of 20 mg daily [95]. The study’s primary objective was to determine SFR rates at weeks 12 and 52. Secondary objectives included colectomy rates, PNR, LOR, and continuity of treatment [95]. Overall, 81 patients (23.7%) achieved SFR at week 12, while 117 (41.1%) achieved the same at week 52, with no significant differences between groups [95]. However, it was found that biologic-naïve patients were more likely to achieve SFR by week 52, both in the ASUC group and the whole cohort. In addition, patients treated with tofacitinib in an early therapeutic phase showed a higher probability of achieving SFR by week 52. The overall colectomy rate was 4.6% at week 12, with ASUC associated with an increased risk of colectomy by week 52, while older age reduced this risk. Compared with international data, both IFX and cyclosporine showed inferior short- and long-term outcomes [95]. During treatment with tofacitinib, clinical activity indices and biochemical markers of inflammation decreased significantly [95]. This study suggests that tofacitinib could be potentially effective as a first-line treatment for biologic-naïve patients, representing a viable option both as salvage therapy and in the early stages of treatment in ASUC.

Tofacitinib has primarily been employed as a salvage treatment, but its use as a first-line therapy has been explored. In a single-centre, double-blind investigation by Singh et al. [96], the use of tofacitinib as initial therapy in ASUC, albeit in combination with corticosteroids, was examined. The study involved 104 adult patients with ASUC, divided into two groups; 53 patients were treated with tofacitinib (10 mg TID) and 51 with placebo while continuing i.v. corticosteroid administration (hydrocortisone 100 mg every 6 h). The primary endpoint, which was the number of patients who responded to treatment according to the Lichtiger index [97] by day 7, was achieved by 83.01% (44 of 53) of the tofacitinib-treated patients, compared with 58.82% (30 of 51) of the placebo-treated patients. Rates of recourse to life-saving therapies, both medical (i.e., IFX) and surgical (i.e., colectomy), were lower in patients treated with tofacitinib on days 7, 30 and 90 [96]. The cumulative risk of needing salvage therapy on day 90 was 13% in patients treated with tofacitinib compared with 38% in placebo patients. In addition, continued treatment with tofacitinib for more than 7 days at reduced doses was observed to be an effective treatment option for maintaining remission [96]. This study also showed that maintenance therapy with tofacitinib reduces the use of life-saving medical or surgical therapies, compared with azathioprine. One possible explanation could be that treatment with the same molecule that led to remission continues to provide favourable effects, whereas azathioprine requires a more extended treatment period (12–16 weeks) to achieve maximum efficacy.

Another report by Komeda et al. [98] demonstrated clinical remission in six of eight bio-naïve ASUC patients treated with tofacitinib. These results also suggest that tofacitinib may be a viable option for ASUC as a first-line treatment. The use of tofacitinib in the first-line setting rather than after IFX may be justified for several reasons. It has a rapid response time (3–5 days), allowing early evaluation of efficacy and decision of whether to continue or switch to IFX; moreover, it has a half-life of 3.2 h, as already mentioned, which is much shorter than IFX (8.1 days), allowing rapid wash-out of the drug in case of failure, reducing the risk of drug overlap in the case of switch.

In a case series, Jena et al. [99] involved four patients admitted with ASUC. Three patients, following tofacitinib administration, achieved improvement in clinical symptoms, thus avoiding colectomy. An analysis of 21 patients with ASUC in whom tofacitinib showed 75% (3/4) effectiveness as first-line therapy, 85.7% (12/14) effectiveness as second-line therapy (steroid failure), and 66.6% (2/3) effectiveness as third-line therapy was performed in the literature review. These results highlight that tofacitinib has significant potential as salvage therapy at different stages of ASUC treatment. The increased effectiveness as a second line might be due to its rapid action, crucial in patients with suboptimal responses to biological or steroid therapies.

However, the decreasing effectiveness of third-line therapies underscores the importance of early intervention. Side effects such as maculopapular rash localised to the trunk and systemic symptoms such as fatigue and body aches have been reported. One particularly critical event was seen in a patient with a recent COVID-19 infection and hypoalbuminemia [99]. This patient developed sudden dyspnoea and hypoxia, likely related to pulmonary embolism [99]. This case highlights the thromboembolic risk associated with tofacitinib, a risk already known for the drug (especially in rheumatoid arthritis patients) and potentially amplified in the context of ASUC.

Therefore, to evaluate the efficacy of tofacitinib in ASUC, it is also necessary to consider potential safety risks, such as infections caused by excessive immune system suppression or thromboembolism, potential side effects of the drug that could be further exacerbated by the prothrombogenic state typical of ASUC [100,101]. Indeed, it has been reported that tofacitinib may be associated with an increased risk of adverse cardiovascular events in patients with rheumatoid arthritis. Regulatory agencies, such as the EMA, warn about the use of the 10 mg BID dose in patients at high risk of thromboembolism [102]. However, these data have not been replicated in patients with UC.

In a 2020 case report from the University of California San Francisco, four patients were hospitalised for ASUC and treated with tofacitinib at a dose of 10 mg BID [103]. All patients had clinical and laboratory improvement and were discharged without needing colectomy and maintenance therapy with tofacitinib. No serious adverse events were noted during the induction or maintenance phases. One patient with a history of VTE remained on chronic anticoagulation therapy during treatment, and no new episodes of thromboembolism developed. This reassuring result suggests that tofacitinib, with caution, can also be used in patients with thromboembolic risk. However, the limited number of patients does not allow definitive conclusions about safety in this high-risk population [103]. It has also been observed that the risk of thrombotic events associated with tofacitinib in UC is similar to that of TNF inhibitors [104]. In the retrospective case–control study by Berinstein et al. [90], a secondary analysis was performed to compare complications between the tofacitinib-treated and control groups. There were no significant differences in the rates of venous thromboembolism, infections, or cardiovascular events in the tofacitinib-treated group compared with controls during the induction phase of treatment or the subsequent 90-day follow-up. Infections included bacterial infections, opportunistic infections, shingles, and COVID-19.

In a systematic review involving 134 patients with ASUC, of whom 42 received an initial dose of 30 mg tofacitinib for a short period, followed by a reduction to 20 mg daily, while the remaining 92 patients were treated with 20 mg tofacitinib, the most common side effect was the occurrence of infections [105]. Sixteen patients (11.9%) developed infections, including two cases of shingles, one recurrent viral pneumonia, five *Clostridioides difficile* (*C. difficile*) infections, one urinary tract infection, one opportunistic infection, and one non-*C. difficile*-related intestinal infection, one Cytomegalovirus colitis, and one case of bacteraemia.

Other side effects reported were nausea and vomiting (one patient, 0.7%), alopecia (one patient, 0.7%), a venous thromboembolic event (one patient, 0.7%), and a maculopapular rash (one patient, 0.7%). In this review, only one patient died; he was an 81-year-old man with type 2 diabetes and chronic obstructive pulmonary disease who had experienced infectious complications 17 days after a colectomy, a procedure that ultimately caused his death [105]. Tofacitinib had been discontinued 17 days before the procedure [105]. However, most patients had previously received other biological treatments and were on concurrent steroid treatment, which is a confounding factor.

Similar rates of serious adverse effects were reported in the previously described study (i.e., the TACOS study [96]) examining the use of tofacitinib as first-line treatment in ASUC combined with corticosteroids. One patient developed haemorrhagic venous infarction and dural venous sinus thrombosis. However, it is important to consider that a high inflammatory burden characteristic of ASUC and concomitant corticosteroid use may have played a role in developing this thrombosis. Although combined treatment results in greater immune suppression, the risk of infection and other adverse effects was like that of the placebo group. Therefore, further studies need to be conducted to more precisely evaluate the efficacy and safety of tofacitinib, both as monotherapy and in combination with other therapies, to define the role and timing of this drug in the treatment guidelines for ASUC. 

Table 1 and Table 2 resume the major case reports and series concerning the tofacitinib profile in ASUC [91,93,98,99,103,106,107,108,109,110,111,112,113,114].

### 3.3. Upadacitinib

Another molecule that has shown promising potential in treating ASUC is upadacitinib. Upadacitinib is a JAK1 selective inhibitor used to treat several chronic inflammatory diseases, including rheumatic, dermatological and gastrointestinal diseases. Through the inhibition of JAK1, Upadacitinib inhibits the phosphorylation of downstream effector proteins, which consequently inhibits the cytokine pathway involved in inflammatory diseases, including UC [115,116].

In a single-centre study, 14 patients with ASUC were identified [117]. The study’s primary endpoint was the clinical response and remission at eight weeks, assessed by the partial Mayo score [118]. In total, 11 patients failed to respond to standard corticosteroid therapy. The Oxford criteria on day 3 determined failure to respond to corticosteroids. Three patients were treated directly with IFX as a second-line treatment for ASUC. All 14 patients failed to respond to prior corticosteroids or biologic treatment and refused colectomy. Based on the efficacy and rapid onset of action of upadacitinib in UC patients, upadacitinib 45 mg/day was administered as salvage therapy. Patients were followed for 8–32 weeks, with all patients completing 8 weeks of endoscopic follow-up. The mean patient age was 41.5 years, with seven (50%) female patients. All patients underwent endoscopic examination at baseline, where 12/14 (85.7%) were E3 (i.e., pancolitis or extensive colitis according to Montreal classification), and 2/14 (14.3%) were E2 (i.e., left-sided colitis according to Montreal classification). At admission, all patients met Truelove and Witts’s criteria for ASUC. The median baseline C-reactive protein was 32.1 mg/L. All 14 patients had used 5-ASA, three had used vedolizumab, two had used adalimumab, and one had used tofacitinib. After 8 and 16 weeks, respectively, one patient underwent colectomy due to poor clinical response, while twelve remained colectomy-free. The surgical resection rate at 8 weeks was 7.1%, while at 16 weeks it was 14.3%. Four patients (28.6%) achieved clinical remission. The mean partial Mayo score decreased from 10.4 to 4.4. Bowel frequency decreased in all patients. ESR levels decreased in 78.6% (11/14) of patients; in particular, the mean absolute value of ESR decreased from 47.1 to 20.3 mm/h. Two patients showed endoscopic remission after 8 weeks of follow-up (14.3%). The mean Mayo endoscopic score decreased from 3 to 1.5 after 8 weeks. Adverse events included herpes simplex virus infection and elevated thrombin time, and the patient was treated with antiviral drugs or anticoagulants without clinical sequelae. Furthermore, no serious infections or venous thromboembolisms occurred.

Also, in a case report by Gilmore et al. [119], the use of upadacitinib as oral rescue therapy for steroid-refractory ASUC in IFX-experienced patients was described. Six patients described in the case had been diagnosed with ASUC according to the Truelove and Love criteria and had been started on conventional therapy with i.v. corticosteroids, with no response. All patients were previously exposed to IFX. Following an inadequate response to i.v. corticosteroids, and after a multidisciplinary discussion, a trial of off-label rescue therapy with upadacitinib 45 mg once a day was proposed. During the follow-up period that lasted 16 weeks, clinical response was observed within 5 days in five patients and on day 7 in the sixth patient. At 16 weeks, five of six patients remained colectomy-free. At 8 weeks, steroid-free clinical remission was achieved in four of six patients (67%), and no significant adverse events were observed throughout the follow-up.

In a multicenter study, 25 patients with ASUC were treated with upadacitinib and intravenous corticosteroids in five different hospitals [120]. The primary endpoint was the rate of colectomy at 90 days. Secondary endpoints included the rate of steroid-free clinical remission, adverse events, and all-cause hospital readmissions. Of the 25 ASUC patients treated with upadacitinib, six (24%) patients underwent colectomy, fifteen (83%) of eighteen experienced steroid-free clinical remission, one (4%) patient experienced a venous thromboembolic event, and five (20%) patients were readmitted. This study highlights that upadacitinib, in combination with intravenous corticosteroids, may be an effective treatment for ASUC.

These encouraging results, although based on small numbers, suggest the potential use of upadacitinib as oral rescue therapy for ASUC, urging the initiation of prospective studies that can validate its effective and safe use in this setting.

### 3.4. Ozanimod

Among the SP1R modulators, the only available evidence for a potential therapeutic role in ASUC pertains to ozanimod. Cohen et al. [121] reported two cases of patients with ASUC treated with ozanimod. The first case involved a 23-year-old male with pancolitis who had previously failed treatment with IFX, vedolizumab, tofacitinib, and ustekinumab. He was treated with intravenous steroids followed by cyclosporine, achieving clinical remission within three days, which was maintained until two weeks after discharge. Ozanimod was introduced, resulting in a favourable response and sustained clinical remission. The second case reported by the authors concerned a 41-year-old female with a history of left-sided UC and previous failure to adalimumab, IFX, vedolizumab, and tofacitinib, who developed steroid-refractory ASUC. She was treated with cyclosporine, achieving clinical remission within four days of initiation. However, cyclosporine was discontinued one week after discharge due to nausea, and ozanimod was introduced, maintaining sustained remission at four months post-discharge. These two cases suggest that ozanimod could potentially be considered as a treatment following cyclosporine discontinuation. However, further evidence is essential.

## 4. Conclusions

**Figure 3:**
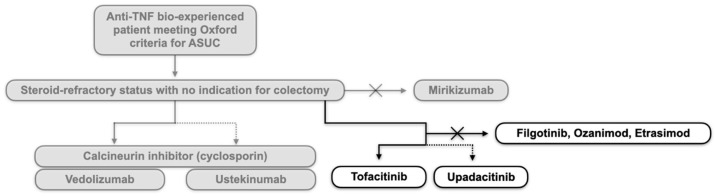
Possible rescue therapies with biologics (including small molecules) to consider in patients with steroid-refractory acute severe ulcerative colitis (ASUC) without indication for surgery and prior exposure to anti-TNF in the disease history. Building on what is already stated in Figure 1 (darker section of the figure), introducing small molecules makes tofacitinib a potential candidate for rescue therapy in ASUC, followed by upadacitinib. Currently, there is no evidence (only anecdotal reports for ozanimod) to justify filgotinib, ozanimod, or etrasimod.

The management of ASUC remains a significant challenge even today for steroid-refractory patients, marking one of the peaks of the post-biologics era. Figure 3 summarises, considering the still strongly needed evidence, the current positioning of small molecules.

There is still a strong need for randomized controlled trials directly targeting patients who meet ASUC criteria to understand which of the advanced therapies currently available for UC are most suitable for an emergency setting, where prompt efficacy and optimal safety are critical parameters for clinical success and the avoidance of potentially fatal complications. Evidence is emerging regarding the potential implementation of small molecules in this context, offering exciting prospects for placing these agents in the ASUC treatment flow charts.

## Figures and Tables

**Table 1:** Major case series reporting efficacy data for tofacitinib in acute severe ulcerative colitis (ASUC).

First Author, Year, Reference	N	Age ^1^	Disease Duration ^2^	Montreal E ^5^	Previous Exposure to Biologics or Small Molecules	ASUC First-Line Treatment with IV Steroids	ASUC Second-Line Treatment	Tofacitinib Dose	Clinical Response Rate (Within 1 Year)	Colectomy Rate (Within 1 Year)
Berinstein et al., 2019 [106]	4	35.5	Ranging from a few weeks to 13 years	E2: 1 (25%) E3: 3 (75%)	1 (25%) IFX 1 (25%) IFX and ADA	Yes (75%)	No	10 mg TID (9 doses); 3 (75%) also received IV steroids	3 (75%)	1 (25%) ^6^
Kotwani et al., 2020 [103]	4	31.5	6	E2: 2 (50%) E3: 2 (50%)	4 (100%) IFX and VDZ	Yes (100%)	No	10 mg BID	4 (100%) ^7^	0 (0%)
Jena et al., 2021 [99]	4	41.7	3.5	E2: 1 (25%) E3: 3 (75%)	No	Yes (100%)	2 (50%) IFX 1 (25%) cyclosporine	10 mg BID	3 (75%)	1 (25%)
Gilmore et al., 2022 [91]	5	22	N.A.	N.A.	3 (60%) IFX 1 (20%) IFX and VDZ	Yes (100%)	2 (40%) IFX	10 mg TID	4 (80%)	2 (10%)
Santos et al., 2022 [93]	2	50	20	E3 2 (100%)	2 (100%) anti-TNF and VDZ	Yes (100%)	No	10 mg BID	2 (100%)	0 (0%)
Xiao et al., 2022 [107]	8	32.5	4	E2: 2 (25%) E3: 6 (75%)	5 (62.5%) IFX	Yes (100%)	3 (37.5%) IFX	5 (62.5%) 10 mg BID; 3 (37.5%) 10 mg BID (3 days) followed by 10 mg BID	5 (62.5%)	3 (37.5%)
Komeda et al., 2023 [98]	8	47.1	2–14 years ^3^	E3 8 (100%)	No	Yes (100%)	No	10 mg bid	6 (75%) ^8^	1 (12.5%)
Eqbal et al., 2023 [108]	11	30.5	3–14 years ^4^	N.A.	6 (54.5%) IFX 3 (27.2%) IFX and VDZ	Yes (100%)	5 (45.4%) IFX	10 mg TID (14 days) followed by 10 mg BID	9 (82%)	2 (18%)
Parra-Izquierdo et al., 2024 [109]	6	34	2.5	E3: 4 (66.7%) E2: 2 (33.3%)	3 (50%) IFX 1 (16.6%) IFX and VDZ	Yes (100%)	2 (33.3%) IFX	4 (66.6%) 10 mg BID; 1 (16.6%) 10 mg tid (3 days) followed by 10 mg BID	6 (100%)	0 (0%)
Ranjan et al., 2024 [110]	4	30.5	4 weeks–4 years	E2: 3 (75%) E3: 1 (25%)	No	Yes (100%)	1 received IFX	1 (25%) 10 mg BID; 3 (75%) 10 mg TID (9 doses) followed by 10 mg BID	3 (75%)	1 (25%)

**Notes**: Where applicable, the percentage relative to the total number of patients included in the series is indicated in parentheses for each parameter. ^1^ The age is reported as the mean of the age values of patients included in the specific case series. ^2^ The disease duration is reported as the mean or as a range when calculating the mean was not possible. ^3^ Within this series, four patients (50%) were at their first diagnosis of UC. Thus, the percentage provided refers to the other half of the patients with a longer-standing prior diagnosis. ^4^ From this percentage, one patient (9.09%) who was at their first diagnosis of UC at the time of the series should be excluded. ^5^ The Montreal classification includes E1 (proctitis), E2 (left-sided colitis), and E3 (extensive colitis/pancolitis). ^6^ One of the three patients underwent colectomy at 6 months due to dysplasia. ^7^ One patient initially responded but later died from respiratory failure due to COVID-19. ^8^ One patient did not respond to TOFA but achieved remission after switching to IFX. *Acronyms*: N: sample size; IV: intravenous; BID: twice a day; TID: three times a day; IFX: infliximab; ADA: adalimumab; VDZ: vedolizumab.

**Table 2:** Major case reports reporting efficacy data for tofacitinib in acute severe ulcerative colitis (ASUC).

First Author, Year, Reference	Age	Disease Duration	Montreal E ^1^	Previous Exposure to Biologics or Small Molecules	ASUC First-Line Treatment with IV Steroids	ASUC Second-Line Treatment	Tofacitinib Dose	Outcome
Sedano et al., 2021 [111]	49	11	E2	IFX	No	IFX dose escalation	30 mg/day	Clinical and endoscopic remission
Yang et al., 2021 [112]	45	5	E3	No	Yes	IFX	10 mg BID and IV cyclosporin 3 mg/Kg/day	Clinical and endoscopic remission
Fortuny Bauzá et al., 2022 [113]	21	1	E3	IFX	No	IFX reinduction	10 mg BID	Clinical response
Girard et al., 2022 [114]	14	First diagnosis	E3	No	Yes	IFX, VDZ, and methotrexate	10 mg BID	Clinical and endoscopic remission

**Notes**: Where applicable, the percentage relative to the total number of patients included in the series is indicated in parentheses for each parameter. ^1^ The Montreal classification includes E1 (proctitis), E2 (left-sided colitis), and E3 (extensive colitis/pancolitis). *Acronyms*: N: sample size; IV: intravenous; BID: twice a day; IFX: infliximab; VDZ: vedolizumab.

## Data Availability

No new data were created or analyzed in this study. Data sharing is not applicable to this article.
